# The chronically inflamed central nervous system provides niches for long-lived plasma cells

**DOI:** 10.1186/s40478-017-0487-8

**Published:** 2017-11-25

**Authors:** Karolin Pollok, Ronja Mothes, Carolin Ulbricht, Alina Liebheit, Jan David Gerken, Sylvia Uhlmann, Friedemann Paul, Raluca Niesner, Helena Radbruch, Anja Erika Hauser

**Affiliations:** 1 Charité - Universitätsmedizin Berlin, Immune Dynamics and Intravital Microscopy, 10117 Berlin, Germany; 2 Deutsches Rheuma-Forschungszentrum Berlin, a Leibniz Institute, Biophysical Analytics, 10117 Berlin, Germany; 3 Charité – Universitätsmedizin Berlin, Experimental and Clinical Research Center and Clinical and Experimental Multiple Sclerosis Research Center, Department of Neurology, and Max Delbrück Center for Molecular Medicine, Berlin, Germany; 4 Dept. of Neuropathology Charité – Universitätsmedizin Berlin, Charitéplatz 1, 10117 Berlin, Germany; 5 Deutsches Rheuma-Forschungszentrum Berlin, a Leibniz Institute, Immune Dynamics, Charitéplatz 1, 10117 Berlin, Germany; 6 Charité – Universitätsmedizin Berlin, NeuroCure Clinical Research Center, NeuroCure Cluster of Excellence, Berlin, Germany

**Keywords:** Multiple sclerosis, Experimental autoimmune encephalomyelitis, B cells, Plasma cells

## Abstract

Although oligoclonal bands in the cerebrospinal fluid have been a hallmark of multiple sclerosis diagnosis for over three decades, the role of antibody-secreting cells in multiple sclerosis remains unclear. T and B cells are critical for multiple sclerosis pathogenesis, but increasing evidence suggests that plasma cells also contribute, through secretion of autoantibodies. Long-lived plasma cells are known to drive various chronic inflammatory conditions as e.g. systemic lupus erythematosus, however, to what extent they are present in autoimmune central nervous system inflammation has not yet been investigated. In brain biopsies from multiple sclerosis patients and other neurological diseases, we could detect non-proliferating plasma cells (CD138^+^Ki67^−^) in the parenchyma. Based on this finding, we hypothesized that long-lived plasma cells can persist in the central nervous system (CNS). In order to test this hypothesis, we adapted the multiple sclerosis mouse model experimental autoimmune encephalomyelitis to generate a B cell memory response. Plasma cells were found in the meninges and the parenchyma of the inflamed spinal cord, surrounded by tissue areas resembling survival niches for these cells, characterized by an up-regulation of chemokines (CXCL12), adhesion molecules (VCAM-1) and survival factors (APRIL and BAFF). In order to determine the lifetime of plasma cells in the chronically inflamed CNS, we labeled the DNA of proliferating cells with 5-ethynyl-2′-deoxyuridine (EdU). Up to five weeks later, we could detect EdU^+^ long-lived plasma cells in the murine CNS. To our knowledge, this is the first study describing non-proliferating plasma cells directly in the target tissue of a chronic inflammation in humans, as well as the first evidence demonstrating the ability of plasma cells to persist in the CNS, and the ability of the chronically inflamed CNS tissue to promote this persistence. Hence, our results suggest that the CNS provides survival niches for long-lived plasma cells, similar to the niches found in other organs. Targeting these cells in the CNS offers new perspectives for treatment of chronic autoimmune neuroinflammatory diseases, especially in patients who do not respond to conventional therapies.

**Electronic supplementary material:**

The online version of this article (10.1186/s40478-017-0487-8) contains supplementary material, which is available to authorized users.

## Introduction

Multiple sclerosis is a chronic autoimmune disease characterized by demyelination and neurodegeneration of the central nervous system (CNS), leading to long-lasting disability. The pathology of multiple sclerosis is not fully understood. Among the hallmarks in the diagnosis of multiple sclerosis is the presence of oligoclonal bands in the cerebrospinal fluid, indicative of certain clones of antibody-producing cells in the CNS compartment [48, 69]. However, the contribution of intracerebral plasma cells to the pathogenesis of multiple sclerosis has not been elucidated, and the relationship between those plasma cells and other plasma cell compartments in the body is not clear. A recent publication reported a correlation between disease activity and the number of autoreactive plasma cells present in the bone marrow, but not in the spleen, using a B cell-dependent experimental autoimmune encephalomyelitis (EAE) model [8].

In other neuroinflammatory diseases, specific targets for autoreactive antibodies have been identified in the CNS, e.g. anti-aquaporin-4 (AQP-4) and anti-N-methyl-D-aspartate receptor (NMDAR) [29, 31]. Plasma cells are found in the chronically inflamed CNS [16, 50, 56], and although oligoclonal bands are known to persist over time in patients [71, 74], few information is available on the life-span of these antibody-producing cells. From other chronic autoimmune conditions like lupus, it is known that long-lived plasma cells contribute to progression of the disease [26]. Plasma cell survival depends on extrinsic factors such as cytokines, for example A proliferation inducing ligand (APRIL) and IL-6, as well as the chemokine CXCL12 [58], with the consequence that long-lived plasma cell survival can only occur in specialized multicellular niches that produce all three factors. In humans, there are several diseases in which the production of pathogenic autoantibodies correlates with disease activity such as in systemic lupus erythematosus [23]. Therapeutic plasma exchange in multiple sclerosis patients with high disease activity provides improvement of disability status [15, 72], and, recently, antibodies targeting axoglial neurofascin have been shown to be capable of worsening disease upon transfer into animal models of EAE [36, 43] and may also play a role in human disease [12]. In marmosets local meningeal T and plasma cell infiltration correlates with subcortical demyelination [30]. In addition, complete depletion of auto-antibodies ameliorates EAE severity in mice immunized with recombinant human myelin oligodendrocyte glycoprotein (rhMOG) peptide [7].

An important role for T and B cells in the pathogenesis of multiple sclerosis is indisputable [24]. A clonal expansion of B cells in the CNS has been proposed to occur in clusters of B cells which resemble follicular structures [25], however, the role of different lymphocyte subsets within the CNS during chronic neuroinflammation is not well investigated. As anti-CD20 treatment has proven to be successful in the treatment of multiple sclerosis [21, 22], B cells have become a focus of research in neuroinflammation [2, 64]. However, various B cell subsets have been shown to act either pro- or anti-inflammatory in multiple sclerosis and EAE, and the proposed involvement of B cells in neuroinflammatory processes includes multiple functions such as antigen presentation, cytokine production and production of autoreactive antibodies [2, 32]. More importantly, plasma cells are not targeted by anti-CD20 therapy [23], and studies with Atacicept, which targets plasma cells, were stopped [19], raising the question which role residual plasma cells play in disease progression.

To investigate the persistence of plasma cells during neuroinflammation, we induced EAE in C57BL/6 mice via injection of rhMOG, a regimen shown to induce a B cell- and antibody-dependent disease [39, 42, 49]. To overcome the monophasic EAE course in C57BL/6 mice and more closely mimic a memory immune response where long-lived plasma cells are generated [20], we boosted the mice with rhMOG, thereby inducing a second active EAE phase, characterized by an increase in MOG-specific antibody titers in serum. Plasma cells were histologically investigated during the course of EAE. Furthermore, to determine the plasma cell lifetime, we performed 5-ethynyl-2′-deoxyuridine (EdU) pulse-chase labeling experiments and were able to identify long-lived plasma cells in the chronically inflamed murine spinal cord. The microenvironment of these long-lived plasma cells resembled the molecular composition of their physiological bone marrow survival niches, with an up-regulation of soluble factors known to attract to and retain plasma cells in their niches, as well as survival factors known to promote plasma cell longevity in the bone marrow. Hence, the CNS -an organ virtually void of peripheral immune cells in healthy individuals- can become a site of persistent immune memory, and may thereby contribute to the chronification of neuroinflammation.

## Material and methods

### Study approval

The study of human material was carried out according to the national ethics guidelines and legal regulations regarding the use of archival material.

All mouse experiments were performed according to institutional guidelines and German Federal laws on animal protection under the licenses G0081/10 and G0076/13 (LaGeSo Berlin).

### Human samples

We investigated archival paraffin-embedded biopsy tissue from patients who had been diagnosed in the Department of Neuropathology, Charité - Universitätsmedizin Berlin with inflammatory demyelination of the central nervous system (CNS) consistent with multiple sclerosis, and with confirmed plasma cell infiltration in their tissue, or with other neurological diseases (OND) (Table 1). All patients with multiple sclerosis fulfilled clinical diagnosis criteria according to McDonald et al. [54]. None of the study authors was involved in decision making with respect to biopsy. Biopsies were taken of white matter lesions. Lesion location of autopsy specimens is detailed in Table 2. Autopsy samples were obtained from the Netherlands Brain Bank (NBB), Netherlands Institute for Neuroscience, Amsterdam. All material was collected from donors from whom written informed consent for a brain autopsy and the use of the material and clinical information for research purposes had been obtained by the NBB.

**Table 1:** Characteristics of the biopsy cases examined

Sample ID	Diagnosis	Presence CD138^+^ Ki67^−^ cells	Age	Treatment	Time from first symptoms to biopsy
MS 1	Acute multiple sclerosis	+	50	none	4 weeks
MS 2	Acute multiple sclerosis	+	22	High dose steroids two weeks prior to biopsy	8 weeks
MS 3	Secondary progressive multiple sclerosis	+	66	Mitoxanthrone	14 years
MS 4	Acute multiple sclerosis	+	58	unknown	9 years
OND 1	Anti-GABA-B receptor encephalitis	+	50	unknown	unknown
OND 2	Whipple’s disease	+	40	unknown	2 years
OND 3	Tuberculosis	+	49	none	unknown
OND 4	Progressive multifocal leukoencephalopathy after immune reconstitution syndrome (PML-IRIS)	+	54	anti-HIV (HAART)	unknown
OND 5	IgG4-related disease	+	45	none	inknown

*MS* multiple sclerosis, *OND* other neurological disease

**Table 2:** Characteristics of the autopsy cases examined

Sample ID	Diagnosis	Presence of CD138^+^ cells	Age died	Treatment	Disease duration	Lesion localization	Lesion activity
MS 5	Secondary progressive multiple sclerosis	+	66	Tamoxifen	21 years	juxtacortical	chronic active
MS 6	Multiple sclerosis	+	44	Morphine and Baclofene	22 years	cortex	chronic active
MS 7	Multiple sclerosis	–	57	Antibiotics, Morphine, Insuline	27 years	cortex	chronic active
MS 8	Multiple sclerosis	–	56	Paracetamol, Cannabis tea, Vitamine D	32 years	cortex and white matter	chronic active
MS 9	Secondary progressive multiple sclerosis	+	58	Morphine and Midazolam	18 years	white matter	chronic active

*MS* multiple sclerosis

### Mice

C57BL/6 J mice were purchased from Charles River and maintained at the DRFZ. C57BL/6 J mice with Th background (expression of MOG-specific B cell receptor [37]) were bred and housed under specific pathogen-free conditions at the animal facility of the Federal Institute for Risk Assessment (BfR, Berlin, Germany). For all in vivo experiments, C57BL/6 J mice were used. Th mice were used only as donors for serum to assemble a relative standard in the ELISA experiments, as a positive control for MOG-specific antibodies.

### Induction and evaluation of experimental autoimmune encephalomyelitis

Mice were 8 to 14 weeks of age at the time of immunization. Experimental autoimmune encephalomyelitis (EAE) was induced by subcutaneous immunization with 60 to 75 μg recombinant human myelin oligodendrocyte glycoprotein protein (rhMOG, AnaSpec) and 800 μg H37Ra (DIFCO Laboratories) emulsified in complete Freund’s adjuvant (DIFCO Laboratories) or 200 μl of recombinant human MOG_1–125_ Hooke-Kit (Hooke Laboratories) followed by two subsequent intraperitoneal injections of 300 ng pertussis toxin (List Biological Laboratories or Hooke Laboratories) at the time of immunization and respectively one or two days later. In some experiments 400 ng pertussis toxin was used, while taking care that controls and testing cohorts received the same amount. Boost was performed four to six weeks after immunization via a second subcutaneous injection with half the amount of the components from the primary EAE induction. Some mice were boosted with complete Freund’s adjuvant and *Mycobacterium tuberculosis* only.

Additionally, some animals received a further intraperitoneal injection of 100 μg ovalbumin (OVA, Sigma-Aldrich) in Alum (Thermo Scientific) at the days of immunization and boost with rhMOG.

Animals were assessed daily for the development of classical EAE signs, which were translated into clinical scores, as follows: 0 = no disease; 0,5 = tail weakness, 1 = complete tail paralysis; 1,5 = tail paralysis plus impaired righting reflex, 2 = partial hind limb paralysis; 3 = complete hind leg paralysis; 4 = complete foreleg paralysis; 5 = moribund.

### Immunohistology of human tissue

The tissue samples were fixed in 4% paraformaldehyde and embedded in paraffin. Antigen retrieval of 3 μm thick deparaffinized sections was performed in 10 mM citrate buffer for 3 min in a pressure cooker. Sections were blocked with PBS/ 5% FCS for 20 min, afterwards the sections were stained with antibodies in PBS/ 5% FCS/ 0.1% Tween20 for minimum 45 min. Following antibodies were used: 4′,6-diamidino-2-phenylindole (DAPI) (Sigma); mouse anti-human-Ki67 (clone Mib-1, DAKO), anti-mouse-Alexa Fluor 546 (polyclonal goat, LifeTechnologies); anti-CD138-FITC (MI15, Biolegend). Sections were mounted with Fluoromount™ Aqueous Mounting Medium (Sigma-Aldrich). Confocal images were generated using a 20×/0.5 numerical aperture (NA) air objective lens on a Zeiss LSM710, provided with Zen 2010 Version 6.0 software. Images were analyzed using Zen 2009 or 2011 Light Edition software (Carl Zeiss MicroImaging).

### In-vivo EdU-pulse chase method

Each mouse received 2,5 mg 5-ethynyl-2′-deoxyuridine (EdU) per day (Invitrogen) and glucose (Braun) per drinking water. Freshly prepared EdU-water was exchanged every two to three days. If rhMOG-immunized mice were unable to drink anymore from the bottle, the same amount of EdU was administered as agarose-gel pad. The treatment after the boost began at day 28 and ended at day 42. Some mice were analyzed on the day of stopping the EdU-feeding (pulse group), others after a three- to five-week chase period (chase group) as indicated in the figure legends.

### Enzyme-linked immunosorbent assay

96-well flat bottom plates (Corning) were coated with 50 μl of a 10 μg/ml anti-mouse Ig (anti-mouse IgM, IgG and IgA, Southern Biotech) or recombinant human MOG_1–125_ protein (AnaSpec) solution overnight at 4 °C. After blocking with PBS/ 3% BSA for 1 h at 37 °C, serum was added, serial dilutions were prepared and plates were incubated for 1 h at 37 °C. For detection, 50 ng biotinylated anti-Ig (anti-mouse IgM, IgG, and IgA, Southern Biotech) were added for 1 h and 50 ng ExtrAvidin®–Alkaline Phosphatase (Sigma-Aldrich) for 20 min both at room temperature. Alkaline Phosphatase Yellow Liquid Substrate (Sigma-Aldrich) was used for detection. As standard, sera from Th mice immunized with recombinant murine MOG_1–125_ (Anaspec) were pooled. Therefore, mice were subcutaneously immunized with 30 to 100 μg recombinant murine MOG (Anaspec) and 800 μg H37Ra (DIFCO Laboratories) emulsified in complete Freund’s adjuvant (DIFCO Laboratories) followed by two subsequent intraperitoneal injections of 200 to 400 ng pertussis toxin (List Biological Laboratories) at the time point of immunization and 2 days later. The sera of Th mice immunized with recombinant mouse MOG were pooled and used as standard for enzyme-linked immunosorbent assay as they have high MOG-specific antibody titers.

All determined concentrations of antibodies were normalized to this standard. Serum from untreated C57BL/6 mice was used as a negative control.

### Preparation of histological sections and microscopy

For tissue fixation, mice were lethally anesthetized and transcardially perfused with ice cold PBS followed by 4% paraformaldehyde. Spinal cord and brain were isolated and further fixed in 4% paraformaldehyde for minimum 4 h up to overnight at 4 °C. Afterwards, organs were prepared for freezing in 15% and 30% sucrose PBS solution each over night at 4 °C. Spinal cord was cut into 8 segments lengthwise, and the spinal cord segments were embedded in O.C.T. compound (Tissue Tek) for cryo conservation and quickly frozen in 2-methylbutan placed on 96% ethanol cooled with dry ice. Samples were stored at −80 °C until preparation of tissue sections. For histological staining, sections of 10 μm were rehydrated in PBS for 20 min and blocked with 10% rat serum (StemCell Technologies) and/or 10% goat serum (Sigma) in PBS/ 1% BSA/ 0,1% Tween20 for 30 min. Afterwards the sections were stained with antibodies in PBS/ 1% BSA/ 0,1% Tween20 for minimum 1 h. Following antibodies and reagents were used: 4′,6-diamidino-2-phenylindole (DAPI) (Sigma); anti-a proliferation inducing ligand (APRIL, clone A3D8, BioLegend), anti-hamster (Armenian)-IgG-FITC (clone Poly4055, BioLegend), anti-mouse-B-cell activating factor (BAFF)-Alexa Fluor 546 (clone 121,808, R&D Systems,DRFZ); anti-mouse-B220-Alexa Fluor 488, 594 and 647 (clone RA3.6B2, DRFZ); anti-CXCL12-digoxygenin (DIG) (clone 79,018, R&D systems, DRFZ); anti-DIG-Alexa Fluor 546 and 594 (DRFZ); anti-GFAP-Alexa Fluor 488 (clone GA5, eBioscience); anit-mouse-Iba1 (polyclonal rabbit, Wako Pure Chemical Industries); anti-rabbit-IgG-Alexa Fluor 594 and 647 (polyclonal donkey, Invitrogen); anti-mouse-IgA-Alexa Fluor 594 (clone 11–44-2, Southern Biotch, DRFZ); anti-mouse-IgG-Alexa Fluor 546 (polyclonal goat, Life Technologies); anti-mouse-IgM-Alexa Fluor 546 and Cy5 (clone M41, DRFZ); anti-mouse-kappa light chain-Alexa Fluor 488, 546, 594 and FITC (clone 187.1, DRFZ); anti-mouse-lambda1 light chain-Alexa Fluor 488, 594 and FITC (clone LS136, DRFZ); anti-laminin (polyclonal rabbit, Sigma); anti-mouse-VCAM-1-Alexa Fluor 488 (clone 6C71, DRFZ). EdU staining was performed using the Click-iT™ EdU Imaging Kits with Alexa Fluor 555, 594 or 647 (Invitrogen). Sections were mounted with Fluoromount™ Aqueous Mounting Medium (Sigma-Aldrich). Confocal images were generated using a 20×/0.5 numerical aperture (NA) air objective lens on a Zeiss LSM710, provided with Zen 2010 Version 6.0 software. Images were analyzed using Zen 2009 or 2011 Light Edition software (Carl Zeiss MicroImaging).

### Histological quantification procedures

The isotype expression of plasma cells as well as the EdU uptake of plasma cells was determined by manual counting of recorded histological images. The number of analyzed plasma cells was indicated in the figure legends.

To quantify the contribution of Iba1- or GFAP-positive cells to BAFF production, we determined the pixel overlay of the 2 channels (BAFF and Iba1 or BAFF and GFAP, respectively) using Fiji Image J software [60]. Subsequently, the ratio of double positive pixels to all BAFF-positive pixels was calculated. Six images from four mice for each time point were analyzed.

### Immunohistochemistry

Immunohistochemical staining of human tissue sections was done with the BenchMark XT ICH/ISH staining module by using the avidin-biotin-complex-method. The following antibodies were used: CD3 (DAKO), CD4 (Zytomed), CD8 (DAKO), CD20 (DAKO), CD68 (DAKO), CD138 (DAKO), Ki67 (DAKO) and MBP (myelin basic protein, A. Menarini Diagnostics). Pictures were taken with a microscope BX50 (Olympus) using a 40× objective lens.

Inflammation was assessed with H&E (Merck) according to standard procedures. Pictures were taken with a compact microscope BZ-9000 (Keyence) using a 10× objective lens. Images were analyzed using Keyence Analyzer software (Keyence).

### Cell preparation and flow cytometry

Mice were lethally anesthetized and transcardially perfused with ice cold PBS. Brain, spinal cord and bone marrow were isolated and single cell solutions prepared. The removed CNS was triturated through a 70 μm mesh and lymphocytes were isolated using 35% Percoll as myelin remained onto the Percoll and could be separated. Directly after lymphocyte isolation, cells were incubated with 20 μg/ml anti-FcgRII/III (clone 2.4G2, DRFZ) in PBS/ 0.2% BSA solution for 10 min at 4 °C. Surface staining was performed using following antibodies: anti-mouse-CD3-FITC (clone 145-2c11, DRFZ); anti-mouse-CD19-Pacific Blue (clone 1D3, DRFZ); anti-mouse-CD4-Alexa Fluor 647 (clone GK1.5, DRFZ); anti-mouse-CD45.2-allophycocyanin (APC) (clone 104, eBioscience); Fixable Viability Dye eFluor®780 (eBioscience). For intracellular staining cells were fixed and permeabilized with BD Cytofix/Cytoperm (BD Bioscience) and stained with anti-mouse-kappa light chain-FITC (clone 187.1, DRFZ) and anti-mouse-lambda1 light chain (clone LS136, DRFZ). EdU staining was performed using the Click-iT™ EdU Imaging Kit with Alexa Fluor 594 (Invitrogen). Stained samples were inquired with a BD LSRFortessa cytometer (BD Biosciences) or a MACSQuant (Miltenyi). Flow cytometric data were analyzed with FlowJo (Tree Star, Inc.) software.

### Statistics

Statistical analysis was performed with GraphPad Prism 6 software, the corresponding tests for significance are specified in the figure legends. *P* values <0.05 were considered significant, no correction for multiple comparisons was made due to the exploratory nature of the study.

## Results

### Non-proliferating plasma cells are present in the CNS of multiple sclerosis patients

**Fig. 1:**
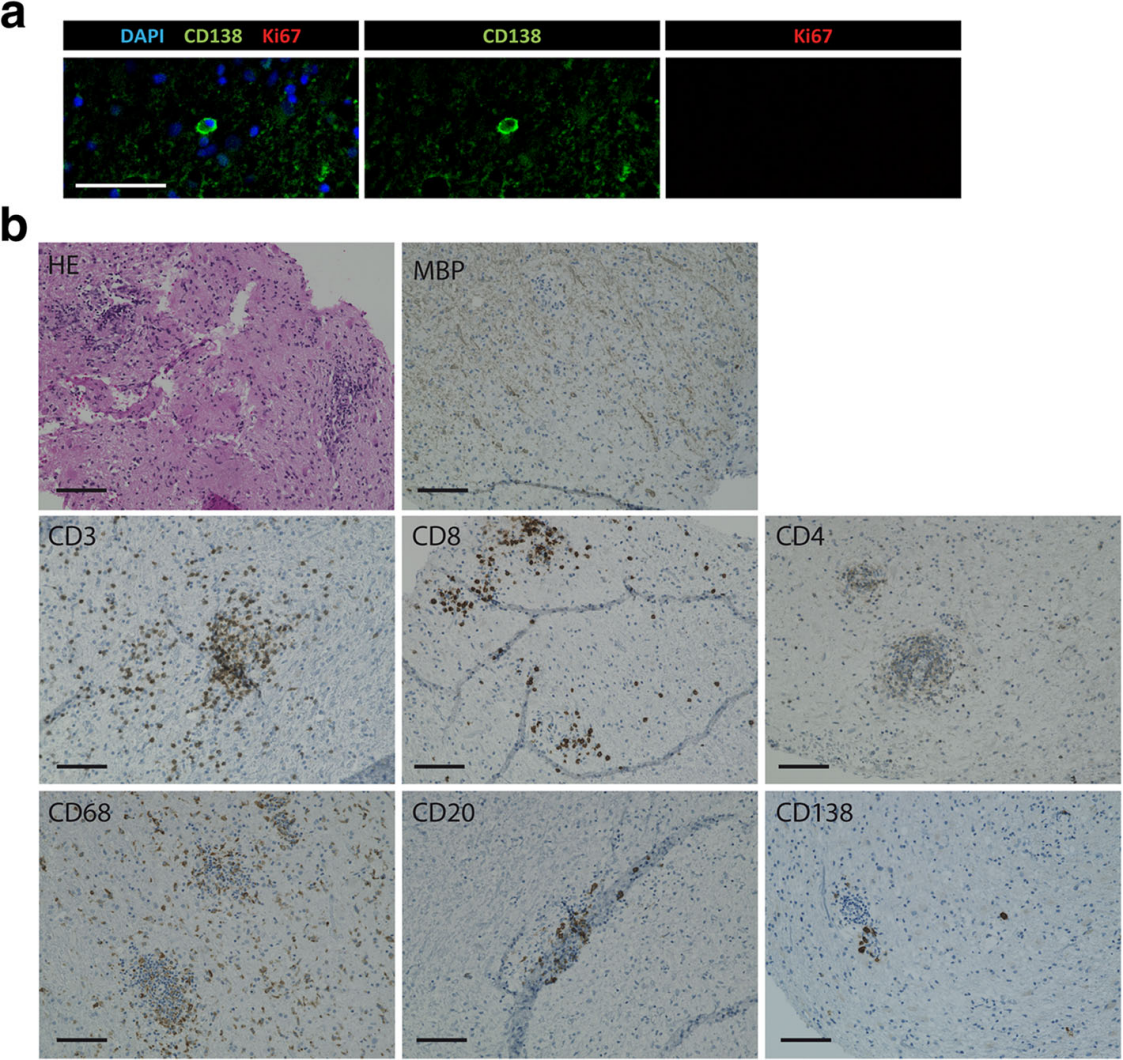
Non-proliferating plasma cells in the brain of multiple sclerosis patients. **a** DAPI (blue), CD138 (green) and Ki67 (red) were stained in the CNS of patient biopsies with multiple sclerosis (*n* = 4). Representative images are shown. Scale bar represents 50 µm. **b** HE, MBP, CD3, CD8, CD4, CD68, CD20 and CD138 staining was performed in the CNS of patients with multiple sclerosis (*n* = 5) in serial slides. Representative images of one patient are shown. Scale bars represent 100 μm.

When analyzing human central nervous system (CNS) tissue from patients with multiple sclerosis (*n* = 7) and other neurological diseases (OND, *n* = 5) (Tables 1 and 2), plasma cells (CD138^+^) were found at various locations, including parenchymal, perivascular and meningeal areas (Fig. 1a, Additional file 1 Fig. S1). The majority of plasma cells in the CNS of multiple sclerosis patients, as well as in other inflammatory brain conditions were non-proliferating, as shown by a lack of the proliferation marker Ki67 (Fig. 1a, Additional file 1 Fig. S1), suggesting that these plasma cells could be long-lived. Representative serial pictures were recorded in order to characterize the immune cell infiltration and demyeliniation in the same patient (Fig. 1b). As there are no methods available specifically addressing the lifetime of plasma cells in the chronically inflamed CNS of humans, we decided to move to a mouse model, allowing us to pulse-label proliferating plasma cells by 5-ethynyl-2′-deoxyuridine (EdU) and thereby tracking their lifetime.

### Adaptation of EAE mouse model to generate a memory response

**Fig. 2:**
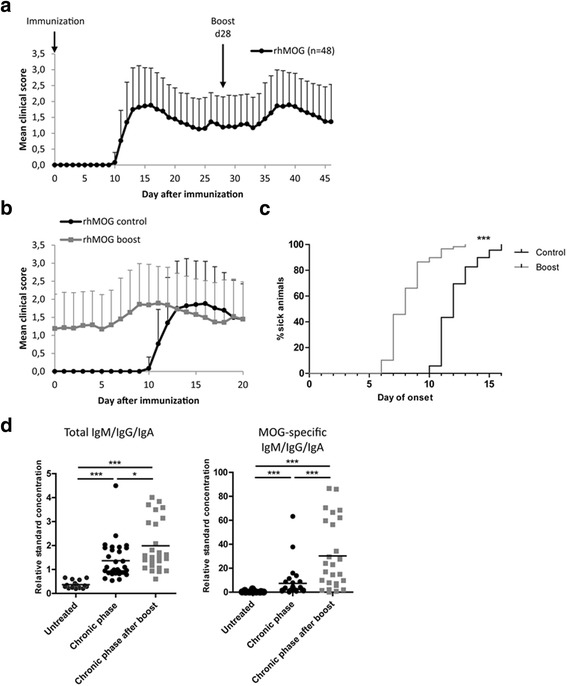
Characterization of EAE-boost model. Mice were immunized and boosted (day 28) with rhMOG. **a** Daily clinical EAE scores are shown. Graph depicts mean value and standard deviation of 48 animals pooled from ten independent experiments. **b** The clinical outcome during the first (control group) and the second (boost group) peak was directly compared. Graph depicts mean value and standard deviation of 48 animals pooled from ten independent experiments. **c** The day of onset was compared between the first (control group) and the second (boost group) peak. The differences between the groups were tested with the Log-Rank (Chi Square) test (***P < 0,001). **d** Graphs demonstrate relative standard concentration of total (left) and MOG-specific immunoglobulin (right) in serum of mice untreated, immunized or boosted, analyzed in the chronic phase. Bars represent mean, each dot represents one mouse pooled from eight (immunized) and five (boost) individual experiments. The statistical differences were tested with the unpaired Mann-Whitney U test (*P < 0,05, ***P < 0,001)

To address this further, we first analyzed plasma cell distribution and longevity in a mouse model of chronic neuroinflammation. Hence, we immunized mice with human recombinant myelin oligodendrocyte glycoprotein (rhMOG), which leads to a known B cell- and antibody-dependent experimental autoimmune encephalomyelitis (EAE) [40, 42, 49]. To avoid the known limitations of C57BL/6 EAE models (only one flare of activity), and account for the fact that long-lived plasma cells are generated in memory responses [20], we modified the immunization protocol to generate a model of relapsing disease: at day 28 after the initial induction, we boosted mice with rhMOG as a secondary immunization in order to induce a memory immune response and a peak after boost. Analyzing the clinical data of the mice after boost, 67% of immunized mice developed a second peak with a mean onset of symptoms at day 36 after EAE induction and concordant maximum disease severity score of 2.1 compared to the first peak (2.1) (Fig. 2a, Table 3). The mean day of clinical onset after boost was four days earlier than during the initial EAE course (Fig. 2b and c, Table 3). Next, we characterized the EAE lesions in our boost model by histology. This analysis revealed massive lymphocyte and macrophage infiltration associated with demyelination in the spinal cord, compared to CNS of control mice immunized with adjuvant only (Additional file 2 Fig. S2).

**Table 3:** Features of EAE development after disease induction and additional boost

	Incidence	Day of onset	Maximum score	Number of animals without relapse after boost	Number of animals with signs occuring after boost only
EAE induction	69/88 (78,4%)	12,3 ± 1,8	2,1 ± 1,2	–	–
Boost	59/88 (67,1%)^a^	36,1 ± 1,6^b^	2,1 ± 1,0^c^	15/88 (17,1%)^d^	9/88 (10,2%)

^a^Increase of scores of ≥0,5

^b^At day 28 boost was performed

^c^Includes clinical scores from animals with constant scores after boost

^d^Animals developed peak after EAE induction, but scores remained stable after boost

To investigate the dependence on antigen for the induction of the second peak, we immunized mice with rhMOG and boosted them with complete Freund’s adjuvant and *Mycobacterium tuberculosis* only. As expected, EAE symptoms did not increase after boost in these mice (data not shown). Furthermore, we analyzed the serum immunoglobulin as a read-out of plasma cell function. Total and MOG-specific immunoglobulin was significantly increased in mice that were immunized and boosted with rhMOG, compared to unimmunized mice (Fig. 2d). Importantly, a strong increase in MOG-specific auto-antibodies was also observed when mice after boost were compared to mice analyzed during the first chronic phase (Fig. 2d, right). Supporting our findings, we observed only a slight increase of total immunoglobulin of mice immunized and boosted with MOG peptide (amino acids 35–55) compared to boosted rhMOG EAE mice, but without the significant increase of MOG-specific auto-antibodies compared to unimmunized mice (Additional file 3 Fig. S3 and Additional file 4 Supplementary Methods), indicating that the rhMOG protein, but not MOG peptide, are capable of anti-MOG autoantibody induction.

### Plasma cells persist in the CNS of EAE mice and become long-lived

**Fig. 3:**
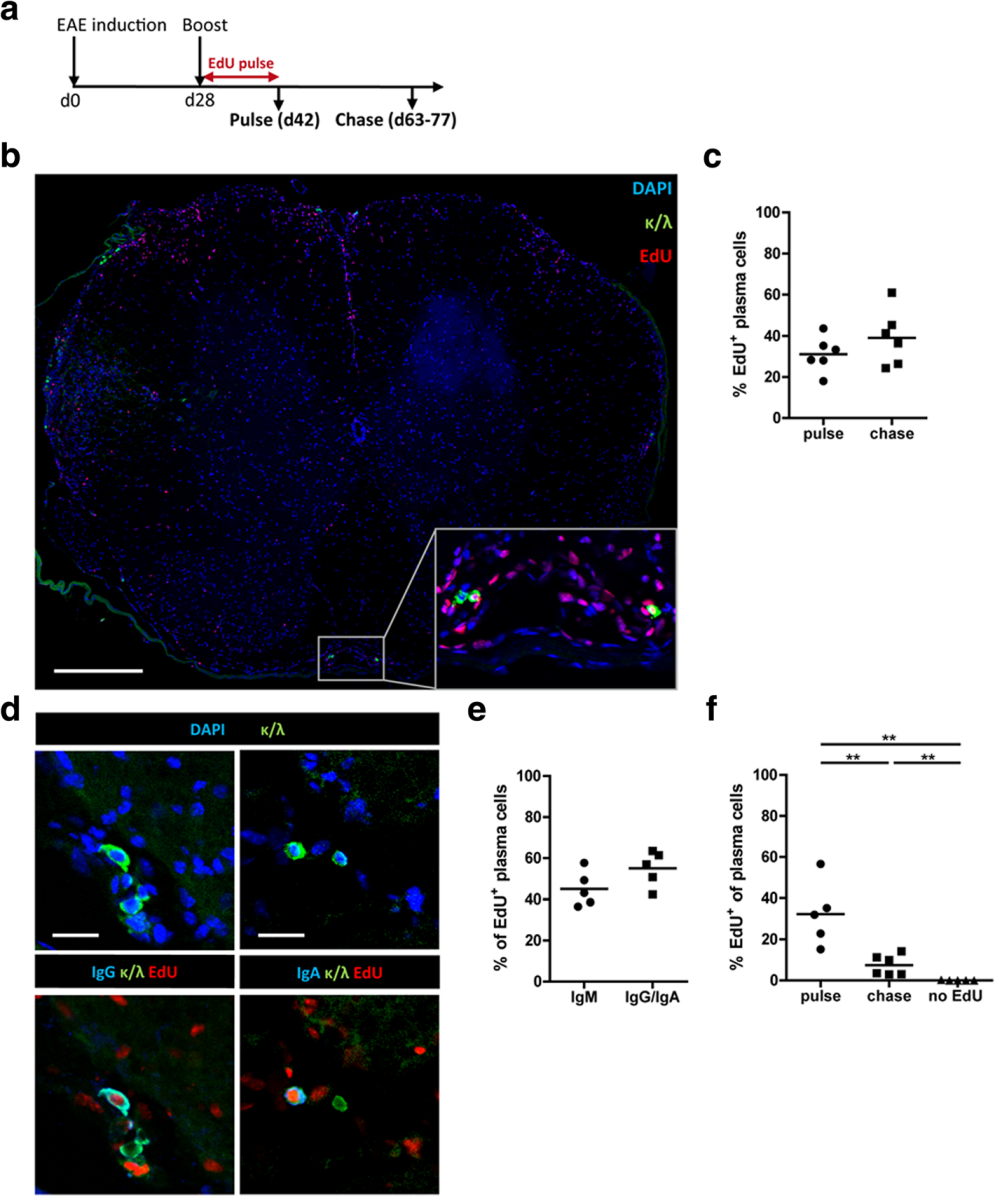
Long-lived plasma cells persist in the chronically inflamed CNS**.** Mice were immunized and boosted (day 28) with rhMOG. **a** The scheme demonstrates the experimental procedure for the EdU pulse-chase experiment, starting after boost. The mice received EdU for 14 days via drinking water. Analysis was performed either directly after stopping EdU-feeding or five to seven weeks after boost. **b** A representative confocal tile scan of a total spinal cord section from day 77 after boost is shown. Signals after immunofluorescence staining of EdU (red), antibody-secreting cells (κ/λ, green) and DAPI (blue) are shown. Data are representative of six mice from three independent experiments. Scale bar of the tile scan represents 200 μm, scale bar of the magnified inset represents 50 μm. **c** The frequency of EdU^+^ plasma cells was determined directly after stopping EdU-feeding (pulse) and after a chase period three to five weeks later. 64 to 362 antibody-secreting cells of each mouse were counted and analyzed manually, mice are pooled from three independent experiments. Bars indicate mean, each data point indicates an individual mouse. **d** Representative confocal microscopy images of inflamed spinal cord of five EAE mice analyzed three to five weeks after stopping EdU-feeding are shown. Antibody-secreting cells (κ/λ, green), EdU (red), DAPI (upper row, blue), IgG (lower panel, left, blue) or IgA (lower panel, right, blue) were stained. Five mice from two independent experiments were analyzed. Scale bars represent 20 μm. **e** The graph demonstrates the frequency of IgM^+^ and IgG^+^/IgA^+^ EdU^+^ plasma cells after the chase period. 52 to 70 EdU^+^ plasma cells of each mouse were counted manually. Bars indicate mean, each data point indicates one individual mouse pooled from two independent experiments. **f** Bone marrow lymphocytes of EAE mice were isolated and analyzed by flow cytometry three to five weeks after stopping EdU-feeding. Viable (eFluor780^−^) lymphocytes (CD45.2^+^) were further analyzed for kappa and afterwards for EdU. Bars indicate mean, each data point indicates one individual mouse. Data of two independent experiments are shown. The significance between the groups was tested with the unpaired Mann-Whitney U test (**P < 0.01)

To investigate if plasma cells can become long-lived directly in the inflamed CNS of EAE mice, dividing cells were labeled during the first two weeks after boost by feeding EdU (Fig. 3a), which incorporates into newly synthesized DNA [59]. Mice were analyzed either directly after stopping EdU-feeding (pulse cohort) or three to five weeks after stopping the EdU-gavage (chase cohort). Histological analysis of CNS sections revealed EdU^+^ as well as EdU^−^ plasma cells in the meninges of the chase cohort (Fig. 3b). Directly after the pulse, approximately one third of total plasma cells were EdU^+^, and the same proportion remained positive after the chase period in the CNS (Fig. 3c), indicating that there were no major changes such as proliferation, migration or apoptosis influencing the CNS-resident plasma cell compartment in the intervening three to five weeks. Interestingly, half of the long-lived plasma cells were class-switched (Fig. 3d and e). These results show for the first time that long-lived plasma cells can persist in the CNS under conditions of chronic inflammation, similar to what has been shown in other inflamed organs as e.g. in the kidney [6, 26]. Notably, we also detected other, kappa/lambda negative, lymphocytes that had taken up EdU in our sections, some of them were CD4^+^ or CD19^+^ as determined by flow cytometry (data not shown) suggesting that not only long-lived plasma cells, but also memory B and T cells, which were generated during the first two weeks after the boost, were found to persist in chronically inflamed CNS. The challenge with rhMOG also resulted in the accumulation of EdU^+^ plasma cells in the bone marrow (Fig. 3f). When comparing the frequency of EdU^+^ plasma cells in both organs, we noticed that the number of plasma cells drops to a greater extent in the bone marrow than in the CNS. Although oligoclonal bands are a key criterion for the diagnosis of multiple sclerosis, the specificity of plasma cells in the CNS is largely unknown. In another neuroinflammatory disorder, anti-N-methyl-D-aspartate receptor (NMDAR) encephalitis, B cells and antibody-secreting cells in the CNS were found to have B cell receptor specificities which recognize CNS structures, as well as B cells with other specificities [31].

**Fig. 4:**
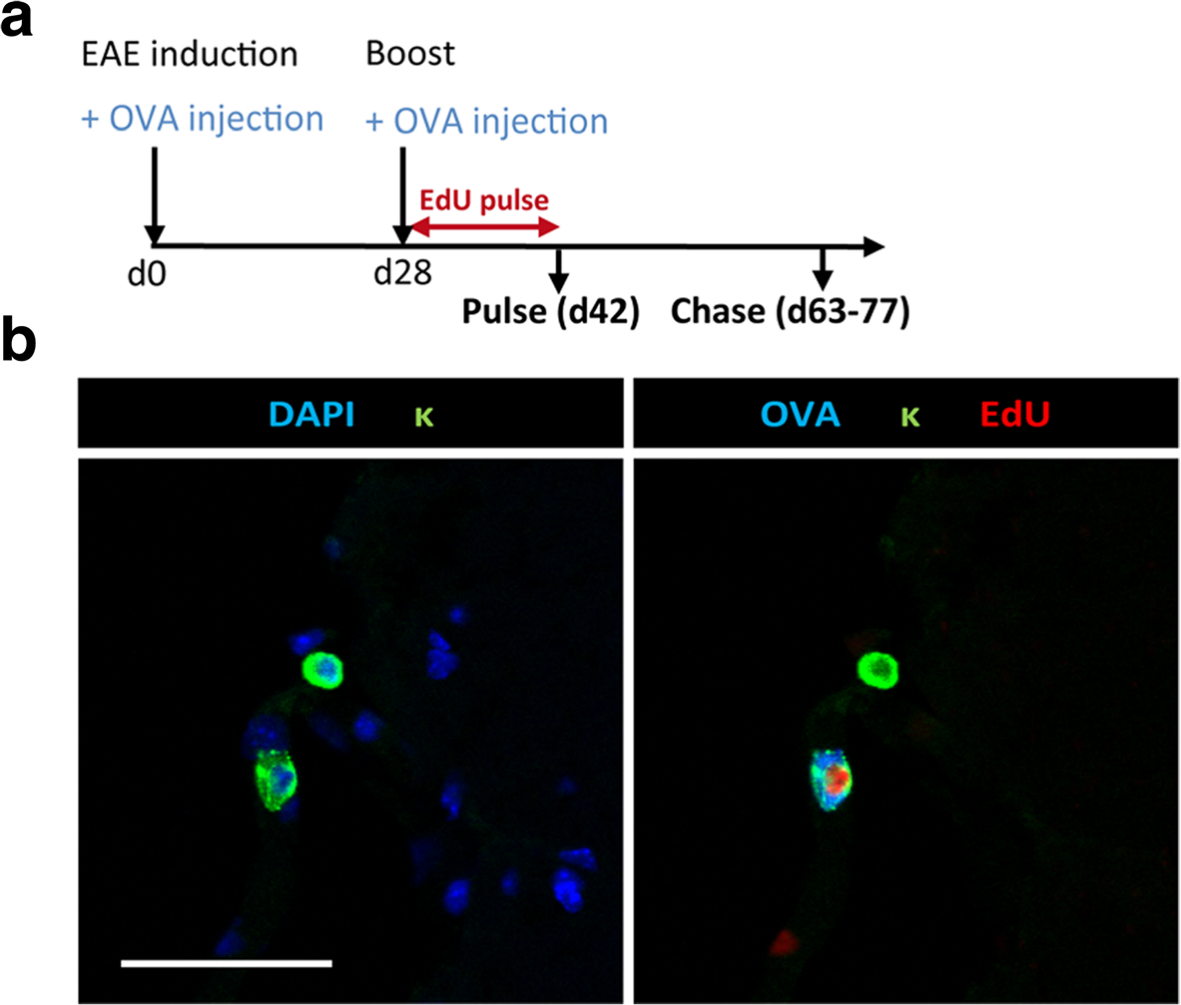
Long-lived plasma cells with non-neuronal or non-self specificities for neuroinflammation persist in the chronically inflamed CNS only to a very low extent. **a** The scheme demonstrates the experimental procedure for EdU pulse-chase experiment starting after boost with additional application of ovalbumin (OVA). The mice were immunized and boosted with rhMOG and OVA. Afterwards, they received EdU for 14 days via drinking water. Analysis was performed directly after stopping the EdU-feeding or five weeks after boost. **b** A representative confocal image of spinal cord from day 42 after boost is shown. Signals after immunofluorescence staining of antibody-secreting cells (κ, green), DAPI (left, blue), EdU (red) and OVA (right, blue) are shown. Data of four mice pooled from two independent experiments are shown. Scale bar scan represents 50 μm

In order to test for the specificity of CNS-resident plasma cells, we modified our EAE protocol by co-challenging the mice with an antigen irrelevant for neuroinflammation (ovalbumin, OVA) at the time of MOG injection (Fig. 4a). We chose OVA because it allows us to detect plasma cells specific for this antigen in the tissue by immunofluorescence microscopy [35]. Indeed, plasma cells containing OVA-specific antibodies, identified by staining with fluorescently tagged OVA, could be detected in the CNS of EAE-diseased mice (Fig. 4b). Notably, OVA-specific plasma cells also had the capability to persist in the chronically inflamed CNS, as indicated by the presence of EdU^+^ OVA-specific plasma cells after the chase period.

### Plasma cell survival niches emerge in the chronically inflamed CNS

**Fig. 5:**
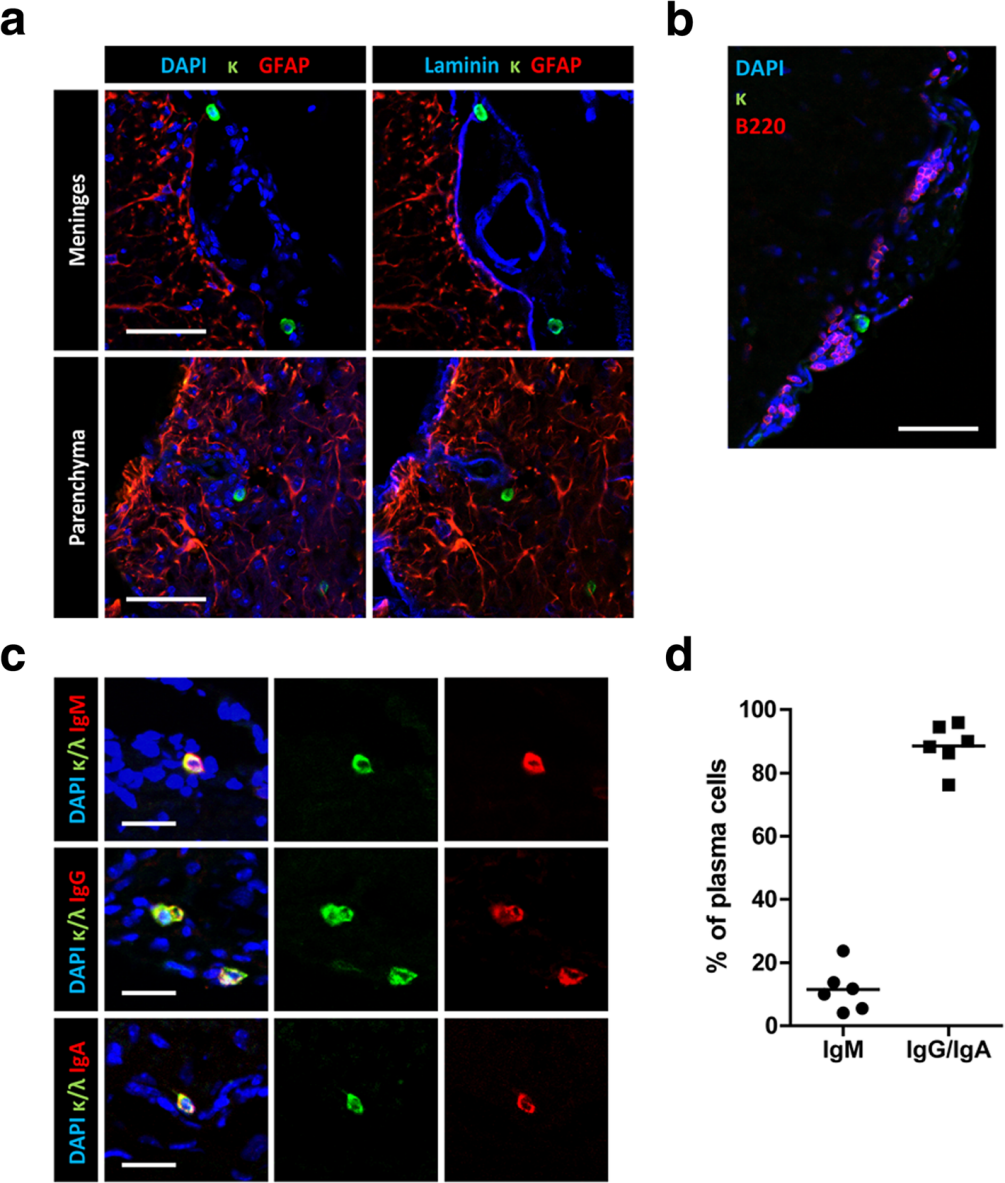
Antibody-secreting cells reside in a supportive microenvironment inflamed mouse CNS during the second peak of EAE. Mice were immunized and boosted (day 28) with rhMOG. Analysis of the spinal cords was performed during the peak after boost. **a** The boundaries of the meninges and the parenchyma are visualized after staining with anti-GFAP (red) and anti-laminin (right, blue) antibodies to determine the relative localization of plasma cells (κ, green) in the inflamed CNS of EAE mice. Representative images of three mice of two independent experiments are shown. Scale bars represent 50 μm. **b** Representative confocal microscopy image of inflamed spinal cord are shown. Antibody-secreting cells (κ, green) are located in the subarachnoid space in the meninges in the proximity of B220^+^ B cells (red). **c** Representative confocal microscopy images of inflamed spinal cord of EAE mice are shown after IgA, IgG and IgM (red) isotype staining of antibody-secreting cells (κ/λ, green). Six mice from three independent experiments were analyzed. Scale bars represent 20 μm. **d** The graph demonstrates the frequency of IgM^+^ or class-switched plasma cells in spinal cord at peak after boost. 51 to 209 antibody-secreting cells each of six mice pooled from three independent experiments were counted und analyzed manually. Bars indicate mean, each data point represents one individual mouse

Next, we further characterized the localization and phenotype of antibody-secreting cells in the chronically inflamed CNS. Plasma cells were found in the meninges and in the perivascular parenchyma (Fig. 5a) in the proximity of B cells (Fig. 5b), confirming previous reports [38, 55]. The majority of the plasma cells were class-switched and only approximately 1/10 (12%) were IgM^+^ (Fig. 5c and d), characteristic of a memory response.

**Fig. 6:**
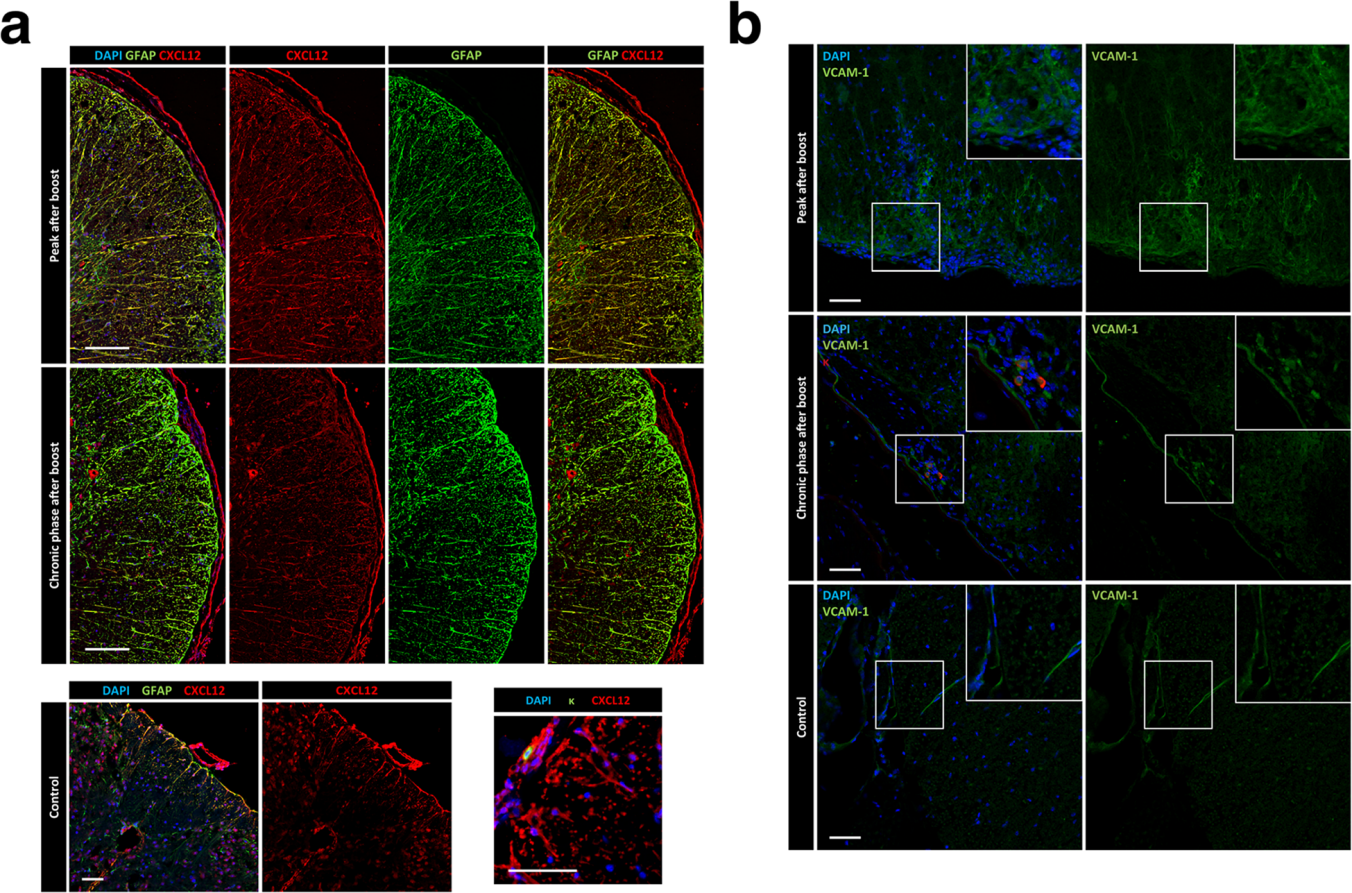
Plasma cell niche signals CXCL12 and VCAM-1 persist in chronically inflamed mouse CNS. Mice were immunized and boosted (day 28) with rhMOG. Analysis of the spinal cords was performed at different time points as indicated. **a** Histology staining was performed with DAPI (blue), anti-CXCL12 (red), anti-GFAP (green) and anti-kappa (κ, right lower panel green) antibody. To determine CXCL12 expression in control CNS, the spinal cord of mice immunized with complete Freund’s adjuvant and *Mycobacterium tuberculosis*, was stained. Only minimal CXCL12 immunoreactivity was detected in the parenchyma. Three to five mice from two independent experiments were analyzed. Scale bars represent 100 μm in the upper panel and 50 μm in the lower panel. **b** DAPI (blue) and VCAM-1 (green) was stained in spinal cord sections of EAE (upper panel) and control mice (lower panel). Three mice from two independent experiments were analyzed per disease stage. Scale bars represent 50 μm

The survival of long-lived plasma cells has been shown to depend on extrinsic factors [58]. By histology, we investigated the presence of these factors in the acute and chronically inflamed CNS. In line with previous reports that showed an increased expression of CXCL12 on blood vessel walls and parenchyma in multiple sclerosis patients [33, 44] and in peptide induced EAE mice [45], we could detect an upregulation of CXCL12 in the lamina glia limitans, the meninges and in the parenchyma at the peak of the disease (Fig. 6a). In addition, we found a persistence of elevated CXCL12 compared to healthy controls in circumscribed tissue regions in the parenchyma and the meninges in the chronic phase (Fig. 6a). The signal partly overlapped with GFAP staining, indicating astrocytes as producers of CXCL12, in line with previous reports [4, 33, 44]. Notably, plasma cells were found to localize in CXCL12^+^ areas (Fig. 6a, right lower panel), supporting the idea that CXCL12 plays a role in attracting plasma cells to inflammatory niches, in addition to its role in mediating plasma cell migration to their physiologic survival niches in the bone marrow [23]. No CXCL12 upregulation was detected when mice were immunized with complete Freund’s adjuvant and *Mycobacterium tuberculosis* (Fig. 6a, left lower panel).

Plasma cell survival niches are characterized by a synergy of multiple molecules which act together in order to support the longevity of their inhabitants. In addition to the chemokine CXCL12, which is involved in attracting plasmablasts into their niches, the adhesion of plasma cells in their niches is thought to be mediated at least partly by VCAM-1, which interacts with the adhesion molecule VLA-4 on plasma cells [14]. In line with published results [17], we could detect an upregulation of VCAM-1 in the CNS of mice affected by EAE at the peak of disease (Fig. 6b). In contrast, a signal for VCAM-1 was restricted to endothelial cells in healthy control mice. In addition, although VCAM-1 expression was reduced during the chronic phase compared to peak (Fig. 6b), we still detected a focal upregulation when compared to healthy mice, and, importantly, plasma cells colocalized at areas of increased VCAM-1 expression, supporting the idea that these areas marked niches in which plasma cells accumulate.

**Fig. 7:**
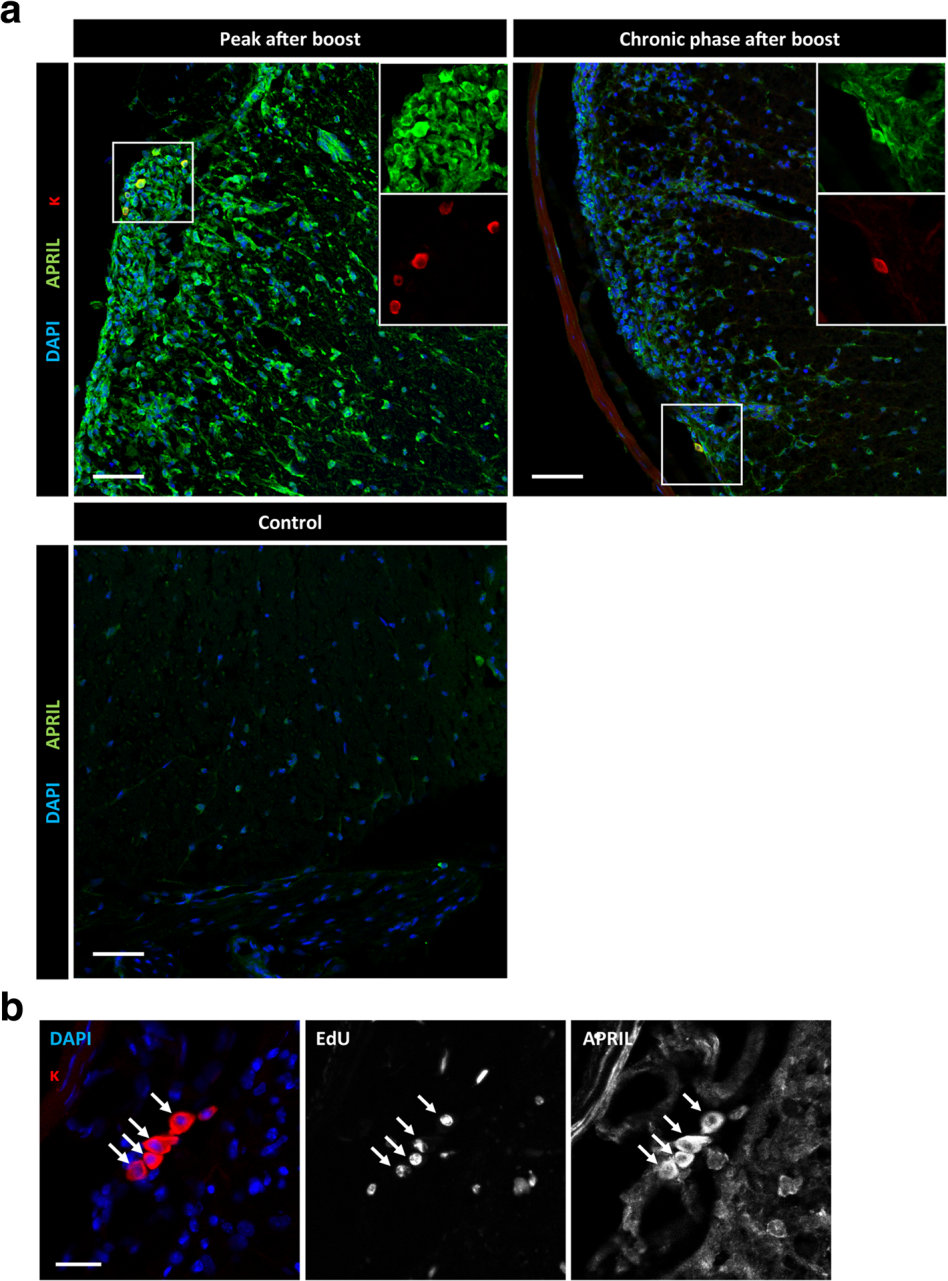
Expression of APRIL in inflamed mouse CNS. Mice were immunized and boosted (day 28) with rhMOG. Analysis of the spinal cords was performed at time points as indicated. **a** The fluorescence signal of DAPI (blue), APRIL (green) and kappa (κ, red) is shown. Magnified insets of the framed area are shown in the right corner. To determine APRIL expression in control CNS, the spinal cord of mice immunized with complete Freund’s adjuvant and *Mycobacterium tuberculosis* was stained (lower panel). Three to four mice from two independent experiments were analyzed. Scale bars represent 50 μm. **b** The mice received EdU for 14 days via drinking water after the boost (day 28). DAPI (blue), kappa (κ, green), EdU and APRIL staining were performed three weeks after stopping the EdU feeding. Four mice from two independent experiments were analyzed. Scale bar represents 20 μm

**Fig. 8:**
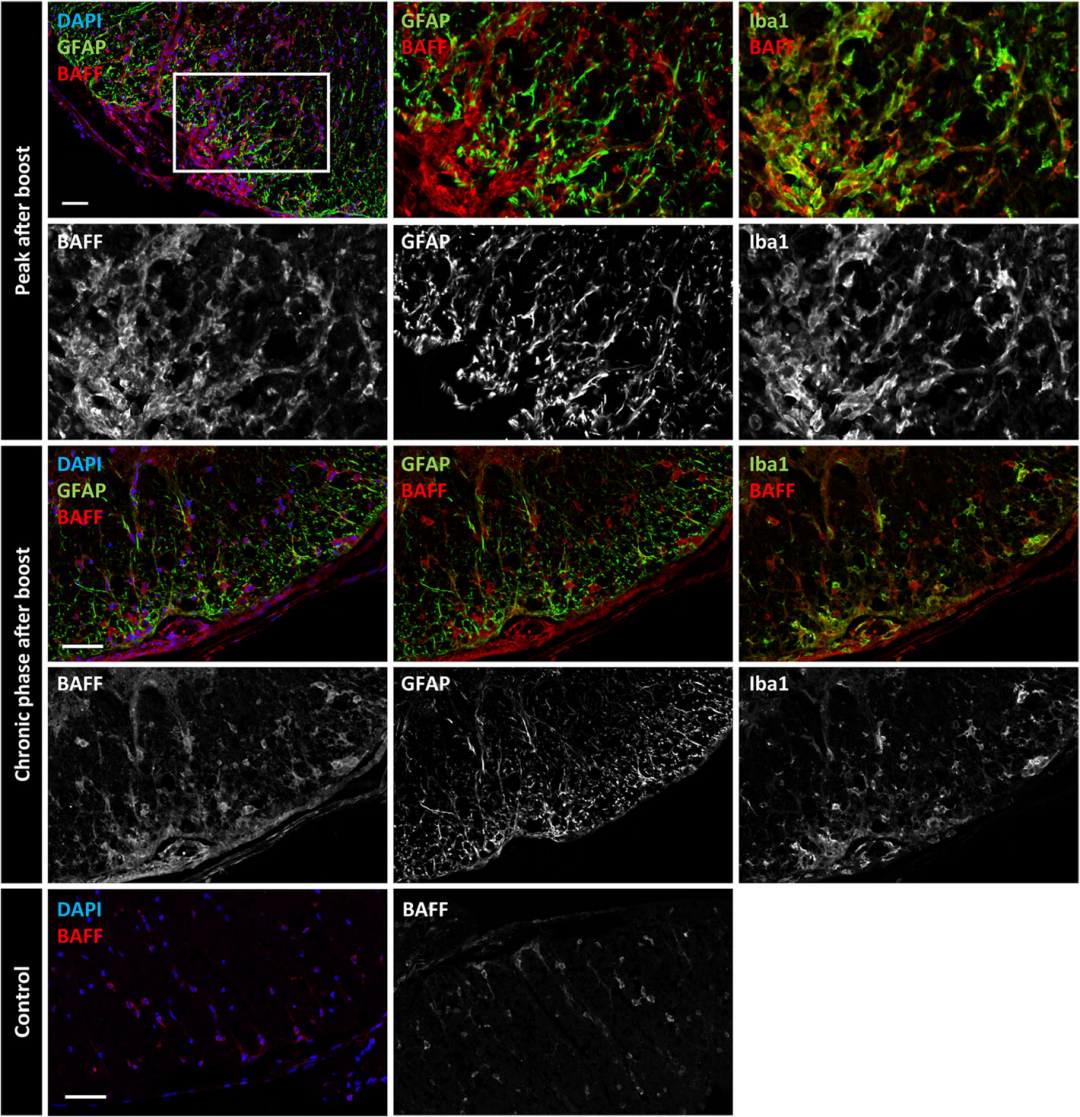
Expression of BAFF in inflamed mouse CNS. Mice were immunized and boosted (day 28) with rhMOG. Analysis of the spinal cords was performed at indicated time points. The fluorescence signal of DAPI, BAFF, GFAP and Iba1 is shown. To determine BAFF expression in control CNS, the spinal cord of mice immunized with complete Freund’s adjuvant and *Mycobacterium tuberculosis* was stained. Scale bars represent 100 μm (peak after boost) and 50 μm (chronic phase after boost and control). Four mice from two to three independent experiments were analyzed for each disease stage

Next, we investigated the presence of survival factors for plasma cells at these locations. Plasma cell survival in the bone marrow has been shown to depend on signaling transmitted by the receptor B cell maturation antigen (BCMA) [3, 47]. Indeed, we were able to detect an increase in the signal of BCMA ligand B-cell activating factor APRIL by immunofluorescence staining of CNS tissue sections from mice at the peak of EAE compared to healthy controls (Fig. 7a, left panel). In addition to APRIL-positive cells morphologically resembling astrocytes, we also detected ovoid-shaped cells that showed a strong positivity when stained with an anti-kappa light chain antibody, indicative of plasma cells. These cells remained strongly positive for APRIL, also during the chronic phase (Fig. 7a, right panel). Co-staining for EdU confirmed that long-lived, kappa^+^ cells with plasmacytoid morphology were positive for APRIL in chronic EAE (Fig. 7b). In conclusion, these results show that the plasma cell survival factor APRIL is produced in the inflamed CNS. In addition to astrocytes, also plasma cells themselves may be a source of APRIL in the CNS, consistent with previous findings in other autoimmune models [9]. We could also find an elevated expression of another BCMA ligand, BAFF, in histology during the EAE peak compared to healthy controls (Fig. 8). Iba1 and GFAP staining revealed both microglia and astrocytes as contributors to BAFF production at the peak of disease: Within the cells showing BAFF positivity by histology, microglia and astrocytes contributed both, as about 30% ± 11% of all BAFF^+^ cells were Iba^+^, and 55% ± 2% co-stained for GFAP. They were also the main populations producing BAFF during the chronic phase of EAE (Iba1: 30% ± 12%; GFAP: 53% ± 3%). In addition to the parenchyma, BAFF was also present in meningeal areas, and B cells as well as plasma cells accumulated specifically in these regions (Additional file 5 Fig. S4). Notably, B cells and plasma cells were found to stain postitive for BAFF in these regions.

Taken together, these findings reveal CNS-resident cells as well as B-lineage cells to stain positive for the BCMA ligand BAFF. Thus, we suggest that both CNS-resident and blood-derived cells support the survival of plasma cells in inflammatory niches.

## Discussion and conclusions

Although B cells became a focus of multiple sclerosis research in the last decade and cerebrospinal fluid (CSF) oligoclonal bands are a hallmark in the diagnosis of the disease, little is known about the role of plasma cells in its pathogenesis.

During immune responses, B cells in secondary lymphoid organs differentiate into antibody-secreting plasmablasts. Most of these plasmablasts are short-lived and die within a few days [63], however, a fraction of them migrates in a CXCL12-dependent fashion to the bone marrow, where they persist as resident plasma cells, given that they reach their physiologic survival niches in the bone marrow [18, 20, 75]. While the number of plasma cells reaching the bone marrow is rather small in primary responses, memory immune responses boost the number of long-lived plasma cells persisting in this organ [20, 41]. The relapsing character of multiple sclerosis also has traits of a memory immune response as the relapses lead to repetitive exposure of the immune system to (auto)antigens [51].

In autoimmune models, antibody-producing cells accumulate and can become long-lived in inflamed tissue [6, 65], where they may produce protective or pathogenic auto-antibodies [27, 73]. Here, we detected long-lived 5-ethynyl-2′-deoxyuridine (EdU)-positive plasma cells generated in a memory response against MOG in the CNS of mice (Fig. 3). Interestingly, the ability of plasma cells to persist in the CNS was somewhat greater than in the bone marrow, as indicated by the greater loss of EdU^+^ bone marrow plasma cells between the pulse and the chase period (Fig. 3f).

Our finding that ovalbumin (OVA)-specific plasma cells can also persist in the CNS suggests that this persistence is independent of antigen-specificity, similar to what has been demonstrated for plasma cells in the inflamed kidneys of lupus mice [6], and consistent with the finding that plasma cell survival is independent of the presence of their specific antigen [41]. In line with our results, an increase of antibody indices against non-CNS antigens can be found in multiple sclerosis patients, the most prominent is the MRZ reaction, composed of three antibody indices against measles, rubella and varicella zoster virus. Notably, it is used as a diagnostic marker for multiple sclerosis patients as it is absent in other inflammatory neurological diseases [28].

Plasma cell survival has been shown to be independent of antigenic stimuli [41], but rather depends on cytokines such as the BCMA-ligand A proliferation inducing ligand (APRIL) [47, 52], in combination with adhesion-dependent signals [14]. The tumor necrosis factor ligand superfamily receptor B cell maturation antigen (BCMA) on plasma cells is known to induce Mcl-1 as a crucial pro-survival pathway in long-lived plasma cells [52]. In the bone marrow, the main ligand for BCMA on long-lived plasma cells is APRIL, produced by eosinophils [10], which constitute transient inhabitants of the plasma cell survival niches [75]. In addition to APRIL, BCMA also binds B-cell activating factor (BAFF), and redundant roles for BAFF and APRIL in supporting plasma cell survival have been demonstrated [3]. These factors have  been shown to be present at elevated levels in the cerebrospinal fluid of multiple sclerosis patients [34, 70]. In line with this, we could detect elevated levels of these factors at the peak, and also during the chronic phase in the CNS of EAE mice, compared to healthy controls (Figs. 7 and 8).

In addition to BCMA ligands, other factors such present in bone marrow niches, the physiologic site for the maintenance of long-lived plasma cells, contribute to plasma cell survival, among them the chemokine CXCL12 and the adhesion molecule VCAM-1 [5, 58, 67].

It is known that CXCL12 is expressed in inflamed tissue [57]. Moreover, CXCL12 has been previously shown to be expressed in the CNS during both multiple sclerosis and EAE, leading to compartmentalization of immune cell subtypes [62]. Here, we detected antibody-secreting cells in proximity to CXCL12^+^ cells, presumably astrocytes as shown by GFAP staining, during the peak of EAE. The observed production of CXCL12 by astrocytes under inflammatory conditions is consistent with published data [1, 11, 33]. With regard to plasma cells, CXCL12 serves as the main factor which attracts those cells to the bone marrow [18, 20]. CXCL12 is expressed constitutively in high amounts by reticular stromal cells of the bone marrow [66], which are a crucial stable component of plasma cell survival niches [75]. Our finding that CXCL12 production by astrocytes continues in the chronic EAE phase suggests that it is involved in creating a microenvironment supportive of plasma cell persistence in the chronically inflamed CNS.

CXCL12 mediates plasmablast chemotaxis to the survival niches, however, to what extent it retains plasma cells within the niches is unclear. VCAM-1 has previously been shown to support plasma cell survival [5]. In addition, VCAM-1 is present in plasma cell niches in the bone marrow [68] and contributes to plasma cell retention at this site, as antibody-mediated blockade of VLA-4, the receptor for VCAM-1, significantly reduces plasma cell numbers in the bone marrow [14]. In the context of neuroinflammation, VCAM-1 production by astrocytes has been previously associated with the manifestation of neurological disease [17]. The selective accumulation of plasma cells in areas with elevated VCAM-1 expression during chronic EAE (Fig. 6b) supports the hypothesis that plasma cells can be retained in CNS survival niches by the VCAM-1/VLA-4 axis.

In addition to CNS-resident cells such as microglia and astrocytes, we found B cells and plasma cells staining strongly positive for both APRIL and BAFF. Our finding is in line with published results [9], suggesting that B-lineage cells can promote their survival, proliferation and differentiation in an autocrine pathway. Notably, Chu et al. found splenic B cells and plasma cells in a mouse model of systemic lupus erythematosus (SLE) express high levels of BAFF [9], and suggested this as a mechanism for the development of this autoimmune disease. Our findings demonstrate that this mechanism of perpetuation also applies directly in the target organ of inflammation, and suggest that this may be a more general mechanism during B cell-mediated autoimmunity.

Taken together, we identified tissue-resident cells specifically and exclusively present in the CNS, which produce survival niche signals for long-lived plasma cells during chronic neuroinflammation. This is in line with our previous findings that sources of plasma cell survival factors have been shown to differ between tissues, and various tissue-resident cell types contribute to the formation of the niches [35]. Additionally, they support the hypothesis that these niches can be generated de novo in chronic inflammation. Thus, we demonstrate that at least three main factors characteristic for plasma cell survival niches, which have previously been shown to support plasma cell longevity in physiologic sites such as the bone marrow, are induced under inflammatory conditions in the CNS. These factors affect various, crucial functions attributed to these niches, namely the regulation of chemotaxis (CXCL12), adhesion (VCAM-1) and survival (APRIL/ BAFF) of plasma cells, and all three functions are required for the survival of these cells.

Our finding of Ki67^−^CD138^+^ cells in the brain of patients diagnosed with multiple sclerosis, indicates that non-proliferative [46] antibody-secreting cells are also present in the human CNS. In contrast to samples from acute inflammatory responses in the CNS, such as progressive multifocal leukoencephalopathy-immune reconstitution inflammatory syndrome (PML-IRIS) showing at least a subset of proliferating CD138^+^ cells (Additional file 1 Fig. S1), we could not detect plasma cells in all multiple sclerosis tissue samples, most probably due to the fact that the distribution of these cells in the CNS is not homogeneous and the autopsy/biopsy specimens only represent small pieces of the CNS. We do not think that the survival of plasma cells in the CNS is specific for multiple sclerosis, but rather consider it a general mechanism of an adaptive immune response during chronic inflammation, as we could also detect them in other neuroinflammatory conditions as e.g. in anti-GABA-B receptor encephalitis (see Additional file 1 Fig. S1) and they are present in other organs as e.g. in kidneys during lupus nephritis or joints of arthritis patients [23]. Long-lived plasma cells are sessile in the tissue [75], and the presence of a CNS-resident autoantibody-producing plasma cell subset may explain the effect of anti-VLA4 antibody (natalizumab) treatment, which hinders lymphocytes from entering the CNS by blocking the function of α_4_ integrin on the surface of immune cells [61], but may also disrupt plasma cell niches.

Taken together, our findings for the first time demonstrate the persistence of immune cells in the chronically inflamed CNS, within inflammatory niches induced de novo by an immune response. Recently, a crucial role for CNS stromal cells in the recruitment of antiviral CD8^+^ T cells was demonstrated in a mouse model of neurotropic virus infection [13]. These findings led to the concept of fibroblastic stromal niches in the CNS, which are capable of sustaining protective immune cells [53]. Our findings add another facet to the role of the CNS stroma in supporting immune cell accumulation, as we also find that stromal cells are able to produce critical factors for the longevity and persistence of immune cells in a chronic autoimmune setting. These inflammatory niches share crucial characteristics with physiologic survival niches for immune cells, in this case plasma cells, and thereby support the persistence of these cells, irrespective of their specificity, even in tissues which are considered virtually void of peripheral immune cells under healthy conditions. The selective disruption of such survival niches for long-lived plasma cells which continually produce autoreactive antibodies could offer new therapeutic approaches for chronic neuroinflammatory diseases.

## Additional files

Additional file 1: Figure S1. Non-proliferating CD138^+^ cells in the brain of patients with other inflammatory neurological diseases (OND). DAPI (blue), CD138 (green) and Ki67 (red) were stained in the CNS of patient biopsies with other neurological diseases (OND, *n* = 4) as indicated on the left. Representative images are shown. White arrows indicate Ki67^+^ CD138^+^ cells. Scale bars represent 50 μm. (TIFF 14779 kb)

Additional file 2: Figure S2. Extensive lymphocyte infiltration during the peak after boost with rhMOG. To analyze the occurrence of infiltrated lymphocytes in the inflamed CNS, HE staining was performed. Mice immunized and boosted with rhMOG (**a**) were compared to mice immunized only with complete Freund's adjuvant and *Mycobacterium tuberculosis* (**b**). Six mice of three independent experiments were analyzed at peak after boost. Scale bar represent 300 μm. (TIFF 13061 kb)

Additional file 3: Figure S3. Comparison of serological antibody titer after immunization and boost with protein or peptide during chronic phase**.** Graphs demonstrate relative standard concentration of total (left) and MOG-specific immunoglobulin (right) in serum of mice, untreated, immunized and boosted with rhMOG or immunized and boosted with MOG_35–55_, respectively. Bars represent mean, each dot represents one mouse pooled from five (rhMOG) and two (MOG_35–55_) individual experiments. The differences between the groups were tested with the unpaired Mann-Whitney U test (**P < 0,01 ***P < 0,001). (TIFF 8783 kb)

Additional file 4: Supplementary methods. (PDF 133 kb)

Additional file 5: Figure S4. BAFF-positive B cells and plasma cells in the inflamed CNS. Mice were immunized and boosted (day 28) with rhMOG. Analysis of spinal cord was performed during peak after boost. The fluorescence signal of DAPI (blue), BAFF (red), kappa/lambda (κ/λ, upper panel green) and B220 (lower panel green) is shown. A plasma cell is indicated with an arrow. Three mice of two independent experiments were analyzed. Scale bars represent 50 μm. (TIFF 20356 kb)
