# Supplementary figures and images for: The chronically inflamed central nervous system provides niches for long-lived plasma cells
Source: Acta Neuropathol Commun. 2017 Nov 25;5:88. doi: 10.1186/s40478-017-0487-8 (PMC5702095; doi:10.1186/s40478-017-0487-8)

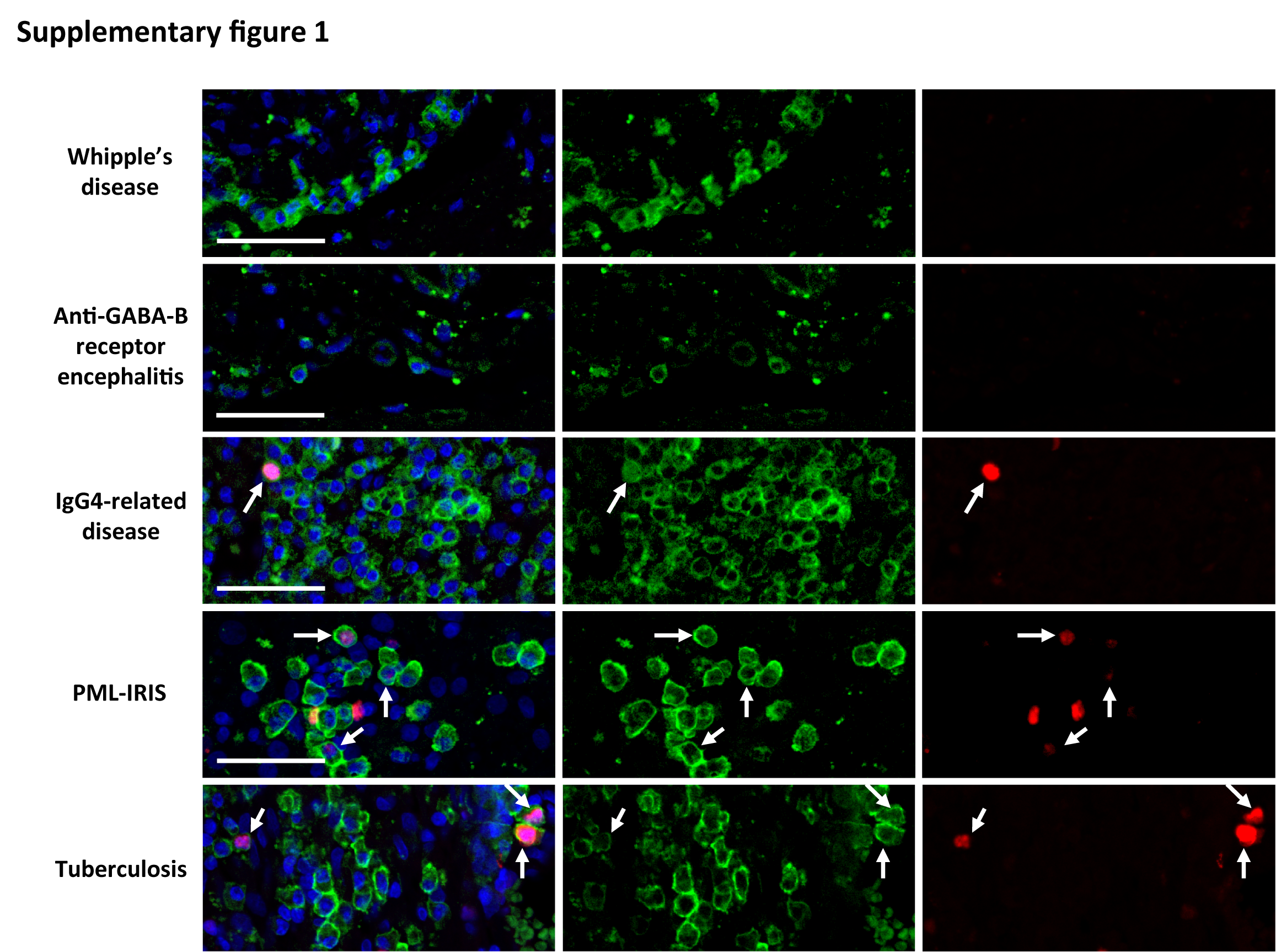

Supplement: Supplementary file 1 — Non-proliferating CD138+ cells in the brain of patients with other inflammatory neurological diseases (OND). DAPI (blue), CD138 (green) and Ki67 (red) were stained in the CNS of patient biopsies with other neurological diseases (OND, n = 4) as indicated on the left. Representative images are shown. White arrows indicate Ki67+ CD138+ cells. Scale bars represent 50 μm. (TIFF 14779 kb) [file 40478_2017_487_MOESM1_ESM.tif]

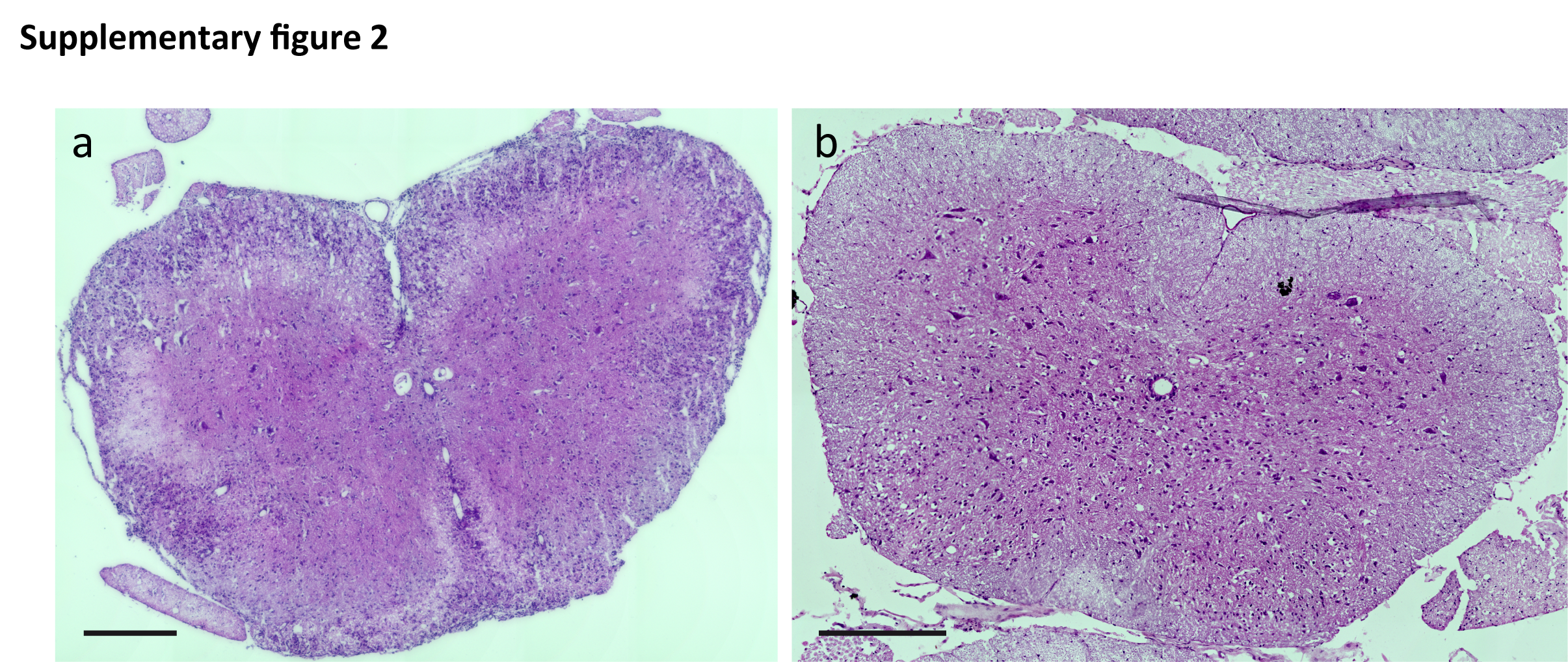

Supplement: Supplementary file 2 — Extensive lymphocyte infiltration during the peak after boost with rhMOG. To analyze the occurrence of infiltrated lymphocytes in the inflamed CNS, HE staining was performed. Mice immunized and boosted with rhMOG (a) were compared to mice immunized only with complete Freund's adjuvant and Mycobacterium tuberculosis (b). Six mice of three independent experiments were analyzed at peak after boost. Scale bar represent 300 μm. (TIFF 13061 kb) [file 40478_2017_487_MOESM2_ESM.tif]

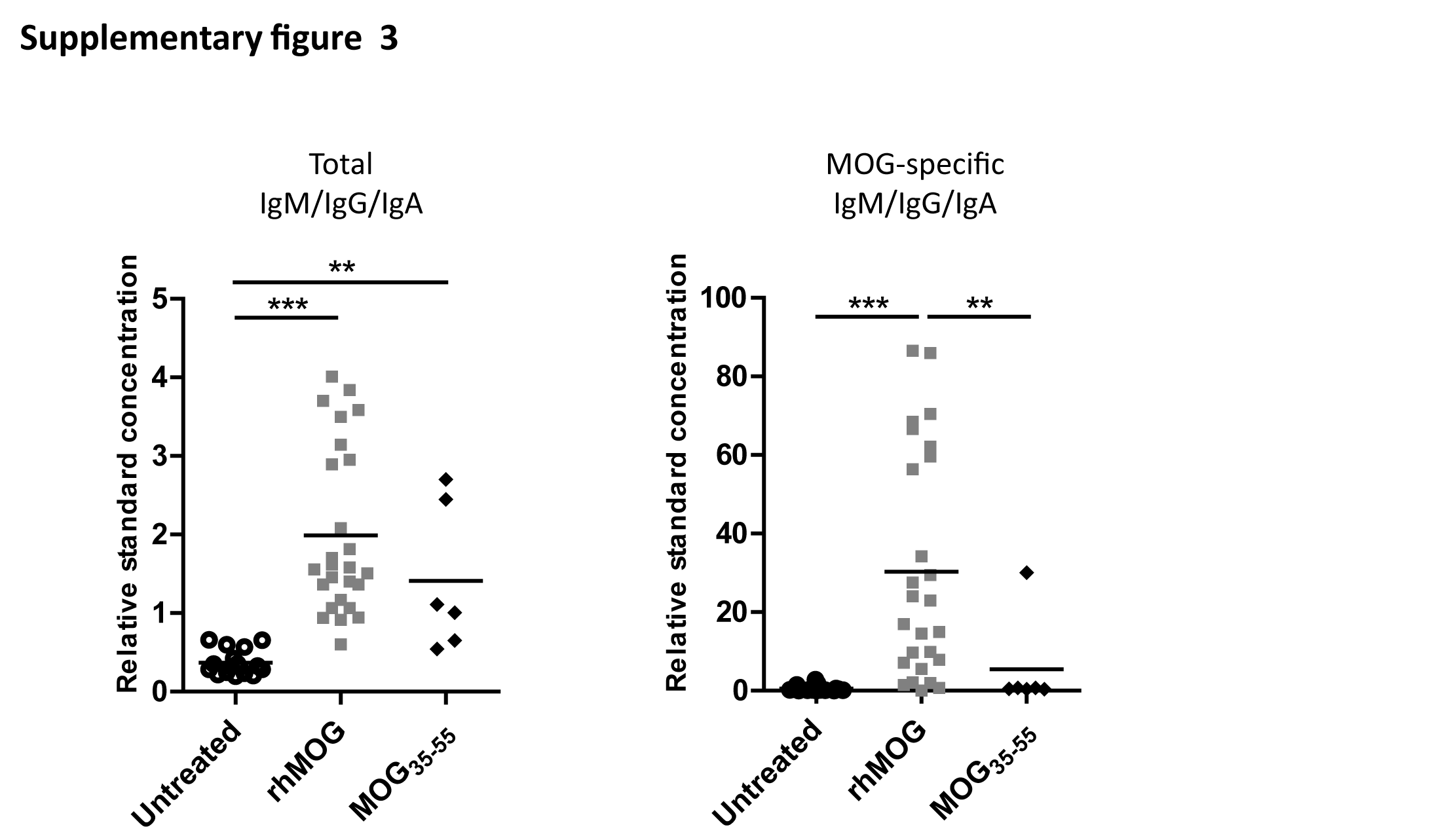

Supplement: Supplementary file 3 — Comparison of serological antibody titer after immunization and boost with protein or peptide during chronic phase. Graphs demonstrate relative standard concentration of total (left) and MOG-specific immunoglobulin (right) in serum of mice, untreated, immunized and boosted with rhMOG or immunized and boosted with MOG35–55, respectively. Bars represent mean, each dot represents one mouse pooled from five (rhMOG) and two (MOG35–55) individual experiments. The differences between the groups were tested with the unpaired Mann-Whitney U test (**P < 0,01 ***P < 0,001). (TIFF 8783 kb) [file 40478_2017_487_MOESM3_ESM.tif]

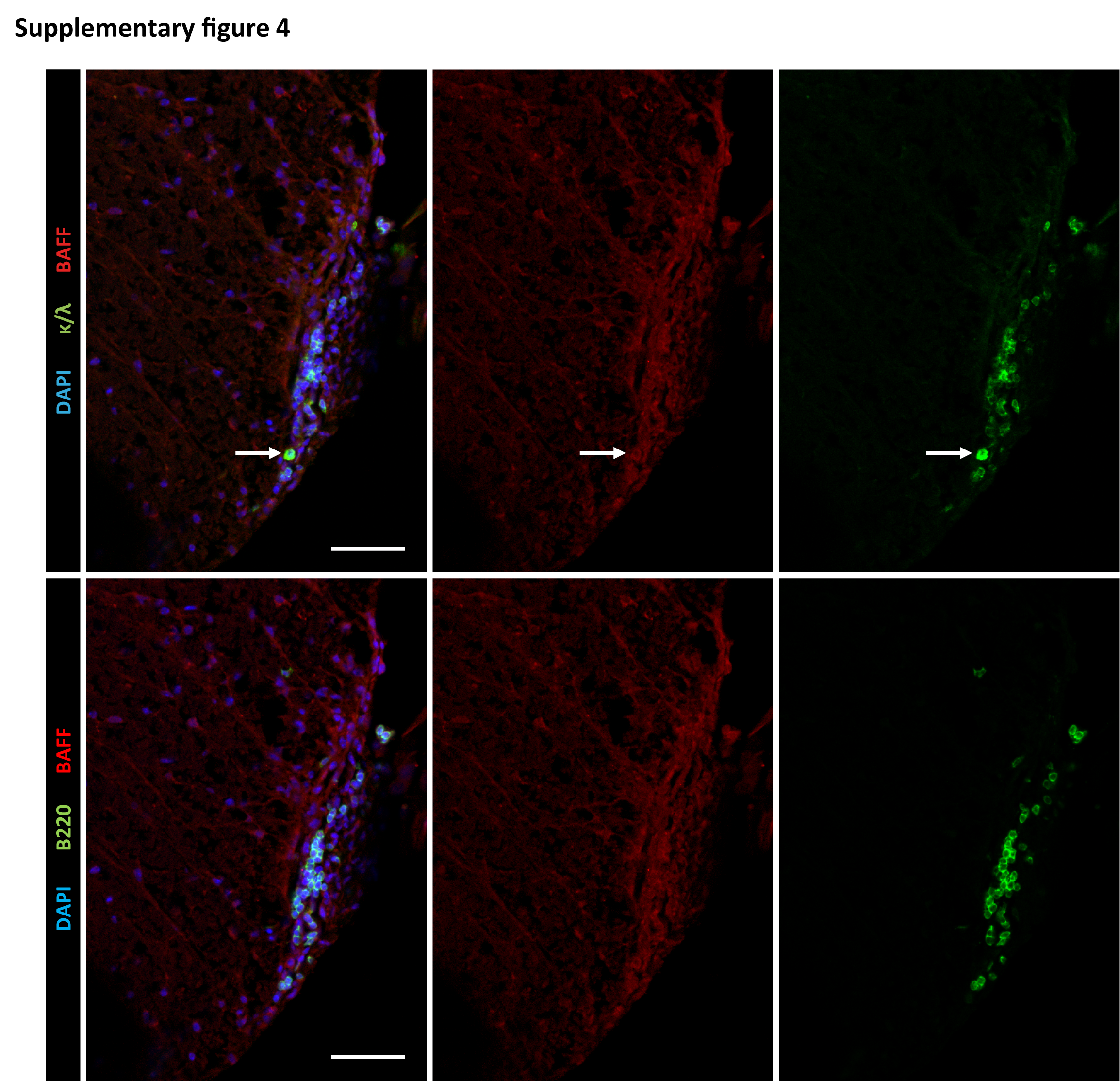

Supplement: Supplementary file 5 — BAFF-positive B cells and plasma cells in the inflamed CNS. Mice were immunized and boosted (day 28) with rhMOG. Analysis of spinal cord was performed during peak after boost. The fluorescence signal of DAPI (blue), BAFF (red), kappa/lambda (κ/λ, upper panel green) and B220 (lower panel green) is shown. A plasma cell is indicated with an arrow. Three mice of two independent experiments were analyzed. Scale bars represent 50 μm. (TIFF 20356 kb) [file 40478_2017_487_MOESM5_ESM.tif]
