# Supplementary material for: The chronically inflamed central nervous system provides niches for long-lived plasma cells
Source: Acta Neuropathol Commun. 2017 Nov 25;5:88. doi: 10.1186/s40478-017-0487-8 (PMC5702095; doi:10.1186/s40478-017-0487-8)
Supplement: Supplementary file 4 — Supplementary methods. (PDF 133 kb) [file 40478_2017_487_MOESM4_ESM.pdf]

Supplementary methods

Induction and evaluation of EAE using $MOG_{35\text{-}55}$

Mice were 8 to 14 weeks of age at immunization. Experimental autoimmune encephalomyelitis (EAE) was induced by subcutaneous immunization with 250 µg $MOG_{35\text{-}55}$ peptide (Pepceuticals) and 800 µg H37Ra (DIFCO Laboratories), emulsified in complete Freund's adjuvant (DIFCO Laboratories) followed by two subsequent intraperitoneal injections of 400 ng pertussis toxin (List Biological Laboratories) at the time point of immunization or two days later. Boost was performed 4 to 6 weeks after immunization via a second subcutaneous injection with half the amount of the components from the primary EAE induction, without further pertussis toxin injection.

Animals were assessed daily for the development of classical EAE signs, which were translated into clinical scores, as follows: 0 = no disease; 0,5 = tail weakness, 1 = complete tail paralysis; 1,5 = tail paralysis plus impaired righting reflex, 2 = partial hind limb paralysis; 3 = complete hind leg paralysis; 4 = complete foreleg paralysis; 5 = moribund.
